# What makes people talk about antibiotics on social media? A retrospective analysis of Twitter use

**DOI:** 10.1093/jac/dku165

**Published:** 2014-05-25

**Authors:** Oliver J. Dyar, Enrique Castro-Sánchez, Alison H. Holmes

**Affiliations:** 1 Medical Education Centre, North Devon District Hospital, Raleigh Park, Barnstaple, Devon EX31 4JB, UK; 2 Centre for Infection Prevention and Management, Imperial College London, Faculty of Medicine, Commonwealth Building, Du Cane Road, London W12 0NN, UK

**Keywords:** antibacterial agents, Internet, Web 2.0

## Abstract

**Objectives:**

Social media has reshaped individual and institutional communication. The unrestricted access to spontaneous views and opinions of society can enrich the evaluation of healthcare interventions. Antimicrobial resistance has been identified as a global threat to health requiring collaboration between clinicians and healthcare users. We sought to explore events and individuals influencing the discourse about antibiotics on Twitter.

**Methods:**

A web-based tool (www.topsy.com) was used to detect daily occurrences of the word ‘antibiotic’ from 24 September 2012 to 23 September 2013 in worldwide Tweets. Activity peaks (message frequency over three times that of baseline) were analysed to identify events leading to the increase.

**Results:**

Of 135 billion messages posted during the study period, 243 000 (0.000002%) referred to ‘antibiotic’. The greatest activity increases appeared after: (i) the UK Chief Medical Officer's (CMO's) declaration of antimicrobial resistance as a national risk (January 2013 and March 2013); (ii) the release of the US CDC's report on antimicrobial resistance (September 2013); and (iii) the US FDA announcement on azithromycin safety concerns (March 2013). The CMO report in March reached an estimated worldwide audience of 20 million users in a single day. However, the frequency of antibiotic Tweets returned to basal levels within 48 h of all four peaks in activity.

**Conclusions:**

Institutional events can rapidly amplify antibiotic discussions on social media, but their short lifespan may hinder their public impact. Multipronged strategies may be required to prolong responses. Developing methods to refine social media monitoring to evaluate the impact and sustainability of societal engagement in the antimicrobial resistance agenda remains essential.

## Introduction

The development of social media and social networks has provided unprecedented communication opportunities between individuals, companies and organizations. It is estimated that almost 60% of all Internet users engage with a social media platform.^1^ The use of these tools has reduced the impact of traditional barriers to communication such as organizational hierarchy or socioeconomic status.^2^ As a reflection of society at large, healthcare has also benefited by the innovation and communication potential released by social media tools. For example, public health commissioners and practitioners have been able to find feedback that has traditionally been difficult to obtain from populations where policies or interventions have been implemented.^3,4^ The use of social media also allows unrestricted access to the opinions and sentiments generated spontaneously by those same populations.

### What is Twitter?

**Figure 1.**
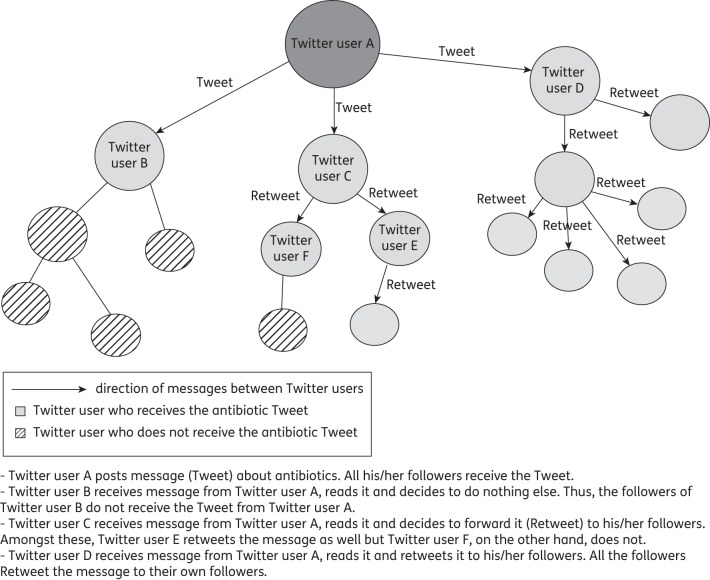
Representation of user interactions on Twitter and flow of Tweets through the network.

Twitter (https://twitter.com) is one of the many different social networks developed over the last decade. This free service allows the mass submission of messages of up to 140 characters, pictures and links (‘Tweets’). These messages are time-stamped and, if enabled, pinpoint the geographical location of the user at the time of posting. Users can be ‘followed’, which means that their messages will be delivered automatically to all their ‘followers’. Followers can endorse messages by ‘favouriting’ or disseminating them (‘Retweeting’) to their own followers (see Figure 1 for an overview of how messages spread on the Twitter platform). It is estimated that in 2013, 18% of the population in the USA used Twitter. However, not all demographic groups are equally represented and current users are mainly young, adult, middle-class males.^5^ Generally, users will post about themselves to share information^6^ and around half of users in the USA use Twitter as a news source.^7^ Some users can have large numbers of followers (hundreds of thousands to millions of people), such as celebrities and news organizations. This direct access helps to explain the popularity of the platform. The use of web analytic measures to rank users according to their online social influence,^8,9^ although debated in terms of accuracy, does allow for the identification of many key influencers within the crowd of individuals and organizations, a useful feature that could be used to maximize the impact of a given health campaign or message.

### Use of Twitter in healthcare and public health

The vast quantity of data generated in real time by users of the service has increasingly been exploited for healthcare and public health analysis. For example, there have been experiences in epidemic intelligence and surveillance epidemiology,^10–12^ including disease activity,^13,14^ understanding public perceptions and attitudes towards public health campaigns and measures such as vaccination,^3,4,15^ and as a tool of public health education and health promotion.^16^ More recently, monitoring and detection of HIV transmission has been achieved using HIV risk-related real-time social media conversations.^17^ Some authors have argued that the potential public health use of Twitter may not just be limited to replacing or complementing current methodologies, but that new sources and types of information could be elicited by studying the wealth of data embedded in social media updates and investigating the networks forged amongst users.^18^

Healthcare institutions do not seem to be taking full advantage of the educational potential of Twitter. A study of social media by UK public health departments reported that whilst 86% had a Twitter account, the publishing of messages was very sporadic and the interactions with audiences were typically limited to the distribution of information. Thus, the potential utilization of this communication channel as a means of engaging in meaningful dialogue with citizens as a way of further promoting public health issues has yet to be realized.^15^ Fostering such engagement is even more important if public health organizations are to successfully address messages expressing negative opinions about health interventions.^19^

Amongst current global public health problems, antimicrobial resistance has been cited by the WHO as one of the top three threats to human health.^20^ The European Centre for Disease Prevention and Control estimated that 25 000 additional deaths occur in EU countries every year due to resistant bacteria.^21^ Given the scale of the threat and the widespread use of antibiotics amongst the general population on a daily basis, efforts are increasingly being made to achieve societal engagement in the antimicrobial resistance agenda. There are annual awareness campaigns in many countries and more recently the UK Chief Medical Officer (CMO) coauthored a short book explaining the tangible dangers posed by resistant bacteria.^22^

Within this context of societal engagement there is potential for social media platforms to be used to raise awareness of growing antimicrobial resistance, to monitor the impact of campaigns and to seek better understanding of the perspectives of individuals on the use of antibiotics. To date, there has been limited analysis of how and when people talk about antibiotics on social media.^23^ Using Twitter as a model platform, we set out to investigate patterns of discussions about antibiotics and to identify drivers that may increase the volume of discussions.

## Methods

A web-based tool (Topsy, www.topsy.com) was used to identify daily occurrences of the word ‘antibiotic’ from 24 September 2012 to 23 September 2013 in public Tweets and Retweets sent from any location worldwide. Topsy is one of the few companies with access to all historic Tweets sent since the launch of Twitter in 2006. Next, we calculated the daily average of antibiotic Tweets and used that average as the baseline of activity. Single day peaks, defined as those with a frequency over three times that of baseline, were identified from a graphical representation of activity generated using Topsy. The individual Tweets contributing to these peaks were analysed to identify the presence of particular events leading to the increase. In addition, information on the geographical distribution of users sending antibiotic Tweets on single day peaks, and on basal days, was sought from the analysis tools within Topsy.

## Results

### Frequency of messages (Tweets and Retweets) about antibiotics

During the 1 year study period, 135 billion messages were published by Twitter users and 243 000 (0.000002%) contained the word ‘antibiotic’. Of these, 203 000 (84%) were original Tweets, whilst 39 000 (16%) were Retweets. On average, 556 Tweets about antibiotics were posted per day. Geographically, the majority of Tweets were submitted from users located in the USA (132 000, 54%), UK (19 000, 8%), Canada (9000, 4%), Malaysia (6000, 2%) and Indonesia (5000, 2%).

### Peaks in Tweets about antibiotics

**Figure 2.**
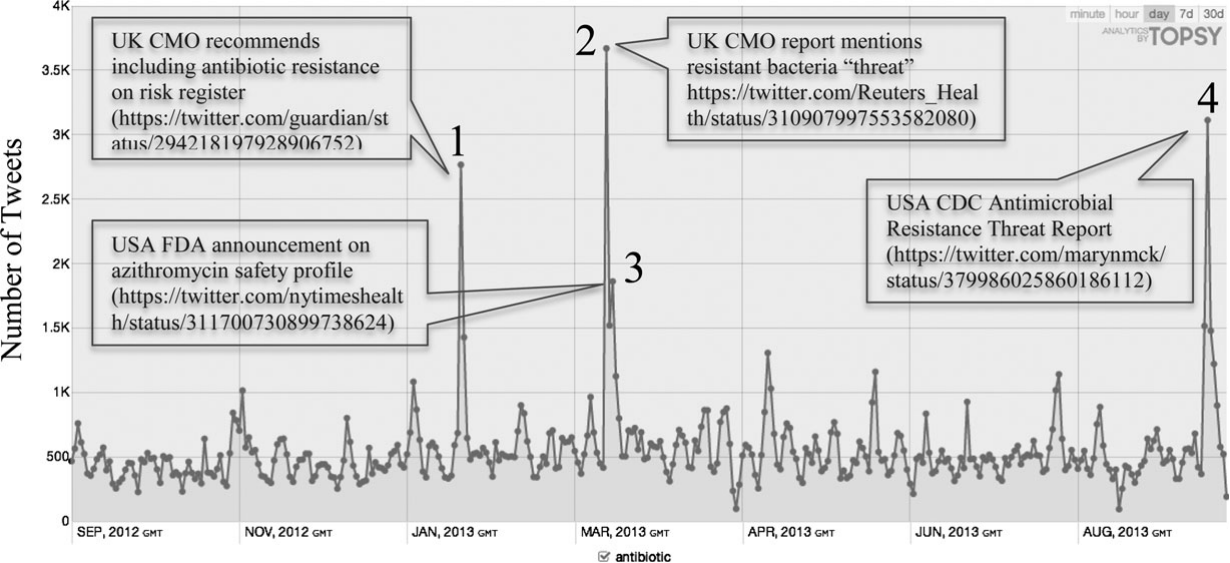
Daily frequency of public Tweets containing the term ‘antibiotic’ over the study period (24 September 2012 to 23 September 2013) with content of peak antibiotic Tweets. Reproduced with permission from Topsy.

The daily occurrences of antibiotic Tweets during the study period are shown in Figure 2. According to our selection threshold, there were four clearly identifiable peaks in activity of antibiotic Tweets (labelled 1–4, Figure 2):

- Peak 1, in January 2013, following the recommendation of the UK CMO to include antibiotic resistance on the national risk register (https://twitter.com/guardian/status/294218197928906752).
- Peak 2, in March 2013, after publication of the UK CMO's annual medical report, which expanded further on the threat of resistant bacteria (https://twitter.com/Reuters_Health/status/310907997553582080).
- Peak 3, in March 2013, as a result of the US FDA's announcement on concerns about the safety profile of azithromycin (https://twitter.com/nytimeshealth/status/311700730899738624).
- Peak 4, in September 2013, occurred after the release of the US CDC's antimicrobial resistance threat report (https://twitter.com/marynmck/status/379986025860186112).

According to Topsy estimates, the CMO's March announcement reached a potential audience of >20 million users in a single day. Such reach was achieved via traditional news outlets such as the British Broadcasting Corporation (www.bbc.co.uk/news) or the *Huffington Post* (www.huffingtonpost.com). Only two individuals featured amongst the top 10 disseminators. For all the peaks identified, and the potential audience reached, the daily number of antibiotic Tweets returned to the baseline frequency within 24–48 h.

## Discussion

Using a web-based tool, we have been able to produce a rapid, brief analysis exploring the presence of the term ‘antibiotic’ on a social media platform with >500 million registered users worldwide. Although messages predominantly originated from the USA and the UK, users in many other countries sent Tweets about antibiotics. The vast majority of these Tweets were original messages, rather than reposts of Tweets from other people, which required users to engage with the content of the message. The daily global discussion about antibiotics did not remain homogeneous in content or volume during the analysis period and we found clearly defined peaks in activity relating to antibiotics. Two of these peaks followed activities by the UK CMO concerning the threat of antimicrobial resistance, which stimulated discussions that reached a large global audience. It has not been possible to tell from our analysis what led to this particular impact compared with other announcements or events.

In all four activity peaks, we found that Tweet activity reached maximal activity within 24 h and returned to the basal levels prior to the peak within 48 h. Thus, although messages about antibiotics spread rapidly in response to events, they do not lead to continued discussions on the Twitter platform at present. It has been suggested that for a certain Tweet, 50% of the total Retweet activity takes place between 4 min and 3 h after the update, suggesting that whilst Tweets can have a short-term impact, a more sustained response would require a multipronged strategy with several Tweets, each with their own large impact.^24^ Other studies suggest that Tweets related to time-sensitive topics or breaking news are more likely to be Retweeted,^25^ highlighting the need for concerted efforts from healthcare organizations to frame the use of Twitter within a cohesive communications strategy and even employing social media managers.

Interestingly, no other antibiotic resistance awareness campaigns resulted in peaks of activity comparable to those seen following the four news announcements discussed above. This was despite the fact that some, such as ‘Get Smart about Antibiotics’ in the USA and the European Antibiotic Awareness campaigns, originated from countries that accounted for the majority of global antibiotic-related messages on Twitter. This would suggest that until now such campaigns have not had a significant impact on this social media platform, which may be due to a lack of engagement with the most influential organizations or individuals on Twitter. Identifying key opinion leaders and gaining their support are essential steps in any public health intervention and appear to be even more crucial when using a network-based communication platform, as people who are more closely connected and interact more often are likely to have greater influence.^26^

As three of the four activity peaks were related to antibiotic resistance, there appears to be a general receptiveness to messages on this topic and future campaigns should take this into consideration. Further, campaigns could look at ways to harness this willingness to discuss antimicrobial resistance into a more sustained dialogue.^15^ Such engagement could create a strong peer-led approach to antibiotic overuse, e.g. encouraging individuals to Tweet when they used recommended self-management approaches rather than asking their healthcare provider for antibiotics. There is some evidence, however, that different types of users generate distinctive discourses on social media and therefore appealing to each audience would need adequate tailoring of messages; organizations and stakeholders in breast cancer, for example, would focus their Twitter updates on fundraising, screening and diagnosis, whilst the public would be significantly more likely to emphasize communal activities and events.^27^ In view of these differences, it is imperative that healthcare organizations evaluate the effectiveness of health promotion and communication theories in different social media platforms.^28^ On the other hand, the prominence of the activity peaks described may reflect the important role that negative sentiments (i.e. unpleasant emotions or attitudes) play when deciding whether to Retweet a message.^29^

As far as we are aware, this is the first study that attempts to characterize events and occurrences driving people to discuss antibiotics on a social media platform. Our study has several limitations. First, by focusing on only one search term (‘antibiotic’) we will have under-reported the total volume of Tweets related to antibiotics, as some will have used other terms such as ‘antimicrobial’ or made direct reference to named antibiotics. We used ‘antibiotic’ as we felt this would be the word most likely to be used by the general population on this subject; indeed, we conducted the same search on Topsy using ‘antimicrobial’ and produced very few results over the study period. Second, our study was limited to terms in the English language only. Third, Topsy uses algorithms to determine whether the message is original or a Retweet and we cannot access these for verification. Fourth, the geographical location of individual Tweets is defined by the location of the user at the time of sending the Tweet and does not necessarily represent their habitual location. Finally, as highlighted in the Introduction, some demographic groups are under-represented amongst Twitter users, so care is required when considering extending our conclusions to other social media platforms and to the general population.

In conclusion, people across the globe talk about antibiotics on social media and free tools can be used rapidly to gain initial insight into discussion topics and triggers for changes in the volume of conversations. Activity increases are global and not localized to the origin of the events. There is a need to understand the contribution and impact of social media tools on public health campaigns and institutional organizations must consider social media within their communications strategy. More evidence is still needed regarding the optimal mix of communication interventions, including the timing of message posting and the individuals best suited to post messages. The development of tools capable of deeper analysis across a broader range of social media platforms may aid assessment of the impact of antibiotic awareness campaigns and promote understanding of opportunities to influence individuals' thoughts about antibiotic resistance.

## Funding

This work was supported by the UK National Institute for Health Research (NIHR) Biomedical Research Centre Funding Scheme at Imperial College (funding number not applicable) and the National Centre for Infection Prevention and Management funded by the UK Clinical Research Council (grant number UKCRC G0800777). A. H. is affiliated with the Imperial Centre for Patient Safety and Service Quality funded by the UK NIHR.

## Transparency declarations

None to declare.

## Author contributions

O. J. D. undertook data acquisition and analysis, drafted and revised the paper and is the guarantor. E. C.-S. drafted and revised the paper. A. H. H. revised the paper. All authors contributed to the development of the concept for the paper and approved the final version.

## References

[1] Statistic Brain http://www.statisticbrain.com/social-networking-statistics.

[2] Keim ME, Noji E (2011). Emergent use of social media: a new age of opportunity for disaster resilience. Am J Disaster Med.

[3] Salathé M, Khandelwal S (2011). Assessing vaccination sentiments with online social media: implications for infectious disease dynamics and control. PLoS Comput Biol.

[4] Lampos V, Lansdall-Welfare T, Araya R (2013). Analysing mood patterns in the United Kingdom through Twitter content. Computing Research Repository.

[5] Duggan M, Brenner J (2013). The demographics of social media users—2012. Pew Internet & American Life Project.

[6] Fox S, Jones S (2009). The social life of health information. Pew Internet & American Life Project.

[7] Holcomb J, Gottfried J, Mitchell A (2013). News use across social media platforms. Pew Research Journalism Project.

[8] T-index (2013). http://www.t-index.co.uk.

[9] Klout (2013). http://www.klout.com/home.

[10] Collier N (2012). Uncovering text mining: a survey of current work on web-based epidemic intelligence. Glob Public Health.

[11] Collier N, Son NT, Nguyen NM (2011). OMG U got flu? Analysis of shared health messages for bio-surveillance. J Biomed Semantics.

[12] Achrekar H, Gandhe A, Lazarus R Predicting flu trends using Twitter data.

[13] Chew C, Eysenbach G (2010). Pandemics in the age of Twitter: content analysis of Tweets during the 2009 H1N1 outbreak. PLoS ONE.

[14] Signorini A, Segre AM, Polgreen PM (2011). The use of Twitter to track levels of disease activity and public concern in the U.S. during the influenza A H1N1 pandemic. PLoS ONE.

[15] Thackeray R, Neiger BL, Smith AK (2012). Adoption and use of social media among public health departments. BMC Public Health.

[16] Vance K, Howe W, Dellavalle RP (2009). Social internet sites as a source of public health information. Dermatol Clin.

[17] Young SD, Rivers C, Lewis B (2014). Methods of using real-time social media technologies for detection and remote monitoring of HIV outcomes. Prev Med.

[18] Paul MJ, Dredze M http://www.cs.jhu.edu/~mdredze/publications/twitter_health_icwsm_11.pdf.

[19] Pereira JA, Quach S, Dao HH (2013). Contagious comments: what was the online buzz about the 2011 Quebec measles outbreak?. PLoS ONE.

[20] WHO (2011). http://www.who.int/world-health-day/2011/en/.

[21] ECDC/EMEA Joint Technical Report (2009). http://www.ecdc.europa.eu/en/publications/Publications/0909_TER_The_Bacterial_Challenge_Time_to_React.pdf.

[22] Davies S, Grant J, Catchpole M (2013). The Drugs Don't Work: A Global Threat.

[23] Scanfeld D, Scanfeld V, Larson EL (2010). Dissemination of health information through social networks: Twitter and antibiotics. Am J Infect Control.

[24] Zaman T, Fox EB, Bradlow ET http://arxiv.org/pdf/1304.6777.pdf.

[25] Naveed N, Gottron T, Kunegis J Bad news travel fast: a content-based analysis of interestingness on Twitter. http://www.journal.webscience.org/435/1/50_paper.pdf.

[26] Bodendorf F, Kaiser C Detecting opinion leaders and trends in online social networks. http://dx.doi.org/10.1145/1651437.1651448.

[27] Thackeray R, Burton SH, Giraud-Carrier C (2013). Using Twitter for breast cancer prevention: an analysis of breast cancer awareness month. BMC Cancer.

[28] Holly Korda H, Itani Z (2013). Harnessing social media for health promotion and behavior change. Health Promot Pract.

[29] Park H, Rodgers S, Stemmle J (2011). Health organizations' use of Facebook for health advertising and promotion. Interactive J Advertising.

